# A Causal and Mediation Analysis of the Comorbidity Between Attention Deficit Hyperactivity Disorder (ADHD) and Autism Spectrum Disorder (ASD)

**DOI:** 10.1007/s10803-017-3083-7

**Published:** 2017-03-02

**Authors:** Elena Sokolova, Anoek M. Oerlemans, Nanda N. Rommelse, Perry Groot, Catharina A. Hartman, Jeffrey C. Glennon, Tom Claassen, Tom Heskes, Jan K. Buitelaar

**Affiliations:** 1 Radboud University, Postbus 9010, 6500 Nijmegen, The Netherlands; 2 Institute for Computing and Information Sciences, Radboud University, Nijmegen, The Netherlands; 3 Department of Psychiatry, Interdisciplinary Center Psychopathology and Emotion Regulation (ICPE), University of Groningen, University Medical Center Groningen, Groningen, The Netherlands; 4 Department of Cognitive Neuroscience, Donders Institute for Brain, Cognition and Behaviour, Radboud University Medical Center, Nijmegen, The Netherlands; 5 Karakter Child and Adolescent Psychiatry University Centre, Nijmegen, The Netherlands; 6 Department of Psychiatry, Donders Institute for Brain, Cognition and Behaviour, Radboud University Medical Center, Nijmegen, The Netherlands

**Keywords:** ADHD, ASD, Inattention, Social interaction, Comorbidity

## Abstract

Autism spectrum disorder (ASD) and Attention-deficit/hyperactivity disorder (ADHD) are often comorbid. The purpose of this study is to explore the relationships between ASD and ADHD symptoms by applying causal modeling. We used a large phenotypic data set of 417 children with ASD and/or ADHD, 562 affected and unaffected siblings, and 414 controls, to infer a structural equation model using a causal discovery algorithm. Three distinct pathways between ASD and ADHD were identified: (1) from impulsivity to difficulties with understanding social information, (2) from hyperactivity to stereotypic, repetitive behavior, (3) a pairwise pathway between inattention, difficulties with understanding social information, and verbal IQ. These findings may inform future studies on understanding the pathophysiological mechanisms behind the overlap between ASD and ADHD.

**Electronic supplementary material:**

The online version of this article (doi:10.1007/s10803-017-3083-7) contains supplementary material, which is available to authorized users.

## Introduction

Autism spectrum disorders (ASD) and attention-deficit/hyperactivity disorder (ADHD) are regarded as distinct disorders in the Diagnostic and Statistical Manual of Mental Disorders (DSM-5). ASD symptoms include impairments in interaction, communication and restricted, stereotyped, and repetitive behavior, whereas ADHD is characterized by symptoms of inattention and hyperactivity/impulsivity (American Psychiatric Association 2013). In previous versions of the DSM, ASD was an exclusion criterion to be diagnosed as having ADHD. As a result, these disorders were studied separately from each other for many years. However, recent research recognizes considerable clinical, genetic, and neuropsychological overlap between ASD and ADHD (Rommelse et al. 2011, 2010a) and within the DSM-5, ADHD can now be diagnosed in conjunction with ASD. Various studies showed that 22–83% of children with ASD have symptoms that satisfy the DSM-IV criteria for ADHD (Ronald et al. 2008; Matson et al. 2013), and vice versa, 30–65% of children with ADHD have clinically significant symptoms of ASD (Clark et al. 1999; Ronald et al. 2008). In clinical practice, it is sometimes difficult to differentiate between ASD and ADHD, partly due to the entanglement of symptom descriptions of both disorders (Luteijn et al. 2000). This might explain why a substantial proportion of children have been alternatively given a diagnosis of one or the other disorder throughout development (Fein et al. 2005). A strong body of twin-, family-, and linkage studies have consistently shown that ASD and ADHD share a portion of their heritable etiology (Lichtenstein et al. 2010b). About 50–72% of the contributing genetic factors overlap between ASD and ADHD (Lichtenstein et al. 2010a; Rommelse et al. 2010b). Furthermore, similar deficits in executive function, social cognition, and motor speed have been linked to both ASD and ADHD (see for an extensive review, Rommelse et al. 2011). Relationships between ASD and ADHD appear to be stronger during certain developmental periods than others, with rather strong ASD/ADHD constellations during adolescence and weaker correlations in early childhood and at adult age. This might be due to that optimal social adaptation and EF skills matter most in adolescence (Hartman et al. 2016).

The main goal of this paper is to investigate what is now needed to resolve this issue of symptom entanglement and alternating diagnoses. Some studies have tried to examine to which degree different symptom domains cluster together, and to which extent these domains are caused by the same genetic and environmental influence (Polderman et al. 2013; Ronald et al. 2014; Taylor et al. 2015). It has been proposed that the association between ASD and ADHD traits is primarily due to shared attention-related problems (inattention and attentional switching capacity), suggesting that biological pathways involving attentional control may be a key factor in unraveling the genetic causes of these disorders (Polderman et al. 2013). However, it is controversial to assume that attentional switching deficits belong solely to ASD and not ADHD. Impulsivity and inattention are often present in individuals with symptoms of ASD and these symptoms have a strong phenotypic and genetic overlap with non-social autistic traits, such as repetitive behavior (Ronald et al. 2014). In contrast, another study showed that genetic overlap was strongest between communication difficulties typical of ASD and ADHD, while repetitive behavior and social difficulties showed only moderate genetic overlap (Taylor et al. 2015). Thus, these studies provide different explanations of comorbidity between ADHD and ASD.

These studies did not assess whether or not the observed links between specific ASD and ADHD traits were due to direct associations or indirect associations. That is, whether or not traits are correlated due to the causal effect of one variable on another or an unobserved common cause for both traits (direct paths) or due to an indirect association mediated via another trait (indirect paths). For example, the finding that social problems were only moderately correlated with hyperactivity, yet strongly correlated with inattention (Ronald et al. 2014), may suggest that the former correlation is explained by an indirect path from social problems to hyperactivity mediated via inattention. Being able to differentiate between direct and indirect paths may greatly improve our understanding of the co-occurrence of ASD and ADHD. In clinical practice it is often unclear what amplifies what, i.e., whether the ADHD related impulsivity is causing the social problems, or reversely, whether the repetitive behaviors are mistaken for hyperactivity. Answering these questions of direction and causation may have significant clinical implications, as it may inform therapeutic interventions.

Standard research methods such as correlation analysis or clustering do not provide the possibility to infer directionality from cross-sectional data. In the current study, the aim is to build a causal model describing the direction of the associations between specific behavioral symptoms of ASD, ADHD, and general factors via a structural equation model (SEM), using the Bayesian Constraint-based Causal Discovery (BCCD) algorithm (Claassen and Heskes 2012). This is an exploratory approach that learns the structure of a SEM from the observed data instead of the more commonly published confirmatory approach that tests *a priori* defined hypothetical networks. The idea of exploratory structure learning algorithms (Pearl 2000) is based on the connection between conditional independencies and causal relationships. Thus, by finding conditional independencies in cross-sectional data, it is possible in particular cases to infer parts of the structure of a SEM and make (preliminary) predictions about causation. BCCD infers the skeleton of the SEM that describes direct associations as well as the direction of effects from data (a detailed description is provided in the Supplementary material). While the skeleton can be accurately inferred from a relatively small sample size, the accurate inference of causal directions requires larger sample sizes (Claassen and Heskes 2012) and the presence of particular patterns to be able to infer the directions. As a second step, standard mediation analysis is applied to test direct or indirect relationships obtained through causal modeling.

In sum, our aim is to explore the relationships between specific ASD and ADHD symptoms by applying causal modeling to a large set of observed data (n = 1393) including children with ADHD and/or ASD, their siblings and control children. Some generic factors are included in our analysis that are known to be associated with ASD and ADHD, namely age, gender, and IQ (Gardener et al. 2009; Mill and Petronis 2008). The current approach primarily determines whether the association between variables is direct, rather than determining the direction of this association, but inferred directions are also included as preliminary hypotheses that should be further tested in independent samples.

## Materials and Methods

### Participants

Participants from two large-scale family-genetic studies, the Biological Origins of Autism (BOA, data collected between 2008 and 2012) study and the Dutch part of the International Multicenter ADHD Genetics (IMAGE data collected between 2004 and 2008) study (van Steijn et al. 2012), were included in the current study. Inclusion criteria for all participants were at least two biological siblings (in case of families: at least one child with a clinical diagnosis of ASD or ADHD), offspring age between 4 and 20 years, European Caucasian descent, offspring IQ ≥70, and no diagnosis of epilepsy, brain disorders, or known genetic disorders, such as Down-syndrome or Fragile-X-syndrome.

All participants were carefully phenotyped for ASD and ADHD using validated and standardized questionnaires and diagnostic interviews. Briefly, both the children already clinically diagnosed with ASD and/or ADHD, their siblings, and the control children were screened for the presence of ASD and ADHD symptoms using the parent- and teacher-reported Social Communication Questionnaire (SCQ)(Rutter et al. 2003) and the parent-, and teacher-reported Conners Rating Scales-Revised (CPRS; CTRS), respectively (Conners 1996). Raw scores of ≥10 on the parent-rated SCQ Total score, ≥ 15 on the teacher-rated SCQ Total score and T-scores ≥63 on the Conners’ DSM-IV Inattention, Hyperactivity-Impulsivity, or Combined scales were considered as clinical. A lower cutoff was used for the parent reported SCQ to avoid false negatives in their undiagnosed offspring (Corsello et al. 2007). All children scoring above cut-off on any of the screening questionnaires underwent full clinical ASD and ADHD assessment, including the Autism Diagnostic Interview-Revised (ADI-R) structured interview for ASD (Le Couteur et al. 2003) and the Parental Account of Childhood Symptoms ADHD subversion (PACS) for ADHD (Taylor 1986). Control children were required to obtain non-clinical scores (i.e., a raw score <10 on the SCQ and T-score <63 on both parent and teacher reported CRS-R DSM-IV scales) in order to be accepted in this study.

The total sample contained 1393 participants, including 586 patients (317 ADHD only, 130 ASD only, and 139 combined ASD+ADHD), 393 unaffected siblings, and 414 controls. Demographics of the study sample are shown in Supplementary Table S1. A more detailed description of participant selection can be found in papers by Steijn et al. and Oerlemans et al. (van Steijn et al. 2012; Oerlemans et al. 2014).

### Measures

To apply causal discovery using the BCCD algorithm, the following variables were selected.

- Age of the participant
- Gender
- Current ADHD symptoms assessed with the parent and teacher reported CRS-R scales.
- Inattention symptoms (CRS DSM-IV inattention subscale).
- Hyperactivity symptoms (hyperactivity items of the CRS DSM-IV hyperactivity/impulsivity subscale).
- Impulsivity symptoms (impulsivity items of the CRS DSM-IV impulsivity subscale).
- Current ASD symptoms assessed with four subscales of the parent-reported Child Social Behavior Questionnaire (CSBQ) (Hartman et al. 2015). A full list of CSBQ items is provided in supplementary material. For clarity we provide a few examples items for each symptom type of CSBQ.
- Reduced contact and social interests (Has little or no need for contact with others, makes little eye contact, etc.)
- Difficulties in understanding social information, referred to as social ineptness further in the text (Takes things literally, e.g., does not understand certain expressions, Does not fully understand what is being said, i.e., tends to miss the point, etc.)
- Fear of/and resistance to changes (Remains clammed up in new situations or if change occurs, panics in new situations or if change occurs, etc.)
- Stereotyped, repetitive behavior (Constantly feels objects, smells objects, etc.)
- Intelligence as measured using the Wechsler Intelligence Scale for Children (WISC-III) or the Wechsler Adult Intelligence Scale (WAIS-III), depending on child’s age (Wechsler 2002, 2000).
- Verbal IQ, prorated by subtests Similarities and Vocabulary.
- Performance IQ, prorated by the subtests Block Design and Picture Completion.

In this study we considered the *raw* data ADHD symptoms for our analyses instead of the T-scale score, since T-scale scores are adjusted for the effect of gender and age. BCCD can model the effect of age and gender into account, and so we avoided unwanted ‘double correction’. Moreover, we separated impulsivity and hyperactivity subscales based on item scores, instead of using the ‘standard’ DSM hyperactivity/impulsivity subscale to examine the effect of each specific trait. For ADHD symptoms, scores assessed by parents and teachers were provided. To increase the reliability of the symptom assessment, for each subject we averaged the symptom scores from parent and teacher. The main two reasons for that are: (1) ADHD is diagnosed when several symptoms are prevalent in at least two or more settings, thus many clinicians find that parent and teacher ratings are helpful in the diagnostic process; (2) parent- and teacher ratings are highly correlated (R = 0.64, p < 0.0001), which makes it difficult to compare them independently. Unfortunately, it was not possible to obtain information about ASD symptoms from a second observer, thus ASD symptoms were assessed only based on parents report.

The CSBQ contains items refer directly to the DSM-IV criteria for autistic disorder, but also represent less severe variations of these criteria as well as ASD-associated problem such as executive function problems and disruptive behavior in social settings (Hartman et al. 2012). We opted for the CSBQ instead of the SCQ to assess ASD symptoms, because we were specifically interested in current behavior, whereas the SCQ mainly refers to behavior at age 4–5 years. Multiple studies have shown that the CSBQ has good psychometric properties with regard to test–retest and interrater reliability, internal consistence of the scales (all reliability indices >0.75), and good criterion validity both for high-functioning children and for children with mild to moderate mental retardation (Hartman et al. 2006; de Bildt et al. 2009; Noordhof et al. 2015; Jaspers et al. 2013; Greaves-Lord et al. 2013). For ASD symptoms only parent scores were provided, so it was not possible to combine them with teacher scores. The reason that only parent-reported ASD symptoms were included in the study is that there is no teacher version of the CSBQ available. The selected WISC-III/WAIS-III subtests are known to correlate between 0.90 and 0.95 with the full-scale IQ (Groth-Marnat 1997).

### Data Analyses

In the first step of the analysis a causal discovery algorithm was used to learn the structure of a SEM, and to formulate hypotheses about direct and indirect relations between variables. Supplementary material describes the link between SEM and causal discovery, provides a description of existing algorithms for causal discovery as well as our motivation for using Bayesian Constraint-based Causal Discovery (BCCD) (Claassen and Heskes 2012). This algorithm infers statements representing causal relationships and estimates the reliability of these statements. The method outputs information about potential interactions between observed variables and does so in two ways: through the skeleton and through orientation. The skeleton describes mediation: two variables are connected if the association between them is not mediated by any other observed variable. Tails and arrows provide information about the direction of the association. The results are visualized through a causal graph by considering statements with reliability higher than 50%. Our method can incorporate prior knowledge about the domain. Here the assumption that gender and participant’s age cannot be caused by any other observed variables in the model was implemented, since chronologically the former are present in the lifespan earlier than the latter.

In the second step of the analysis standard mediation analysis was applied to explicitly check some of the hypotheses generated by our causal analysis. Mediation analysis distinguishes between independent variable, dependent variable, and potential mediators (Baron and Kenny 1986). To test whether the effect of the independent variable is indirect, a regression model was built that aimed to predict the dependent variable from the independent variable and potential mediators. If the regression coefficient was statistically significant for the potential mediators, but not for the independent variable, a conclusion can be made that there was not enough evidence to reject the hypothesis that the effect of the independent variable is indirect.

Note here that the same data is used twice: to generate hypotheses and to test them. Consequently, the reported *p*-values of the mediation test should be treated with care. These *p*-values only indicated the significance as if the specific hypothesis had been coined prior to observing any data. Also note that the data set contains siblings from the same family. To test whether there is an effect of familiality on the resulting causal model, a sensitivity analysis using a subsample of singletons was performed, including only one subject per family. Due to the reduction of the sample size the reliability of the causal links tends to drop. However, if the model is stable, the main links will be preserved in the new model.

## Results

**Fig. 1:**
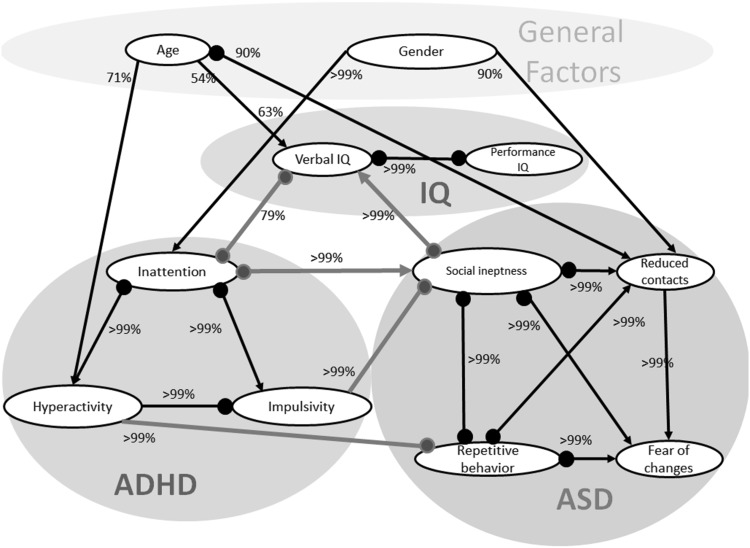
Causal model representing causal relationships between variables in our combined ADHD and ASD data set. Edge directions represent either a causal effect (“*A* → *B*”), an unobserved common cause “*A* ↔ *B*” or a selection bias “*A* − *B*”. Non-identifiable edge directions are marked with a *circle mark* “$\circ$”. Notation “*A* $\circ$ *B*” is either a causal effect “*A* → *B*”, or a selection bias “*A* − *B*”; “*A* $\circ$ → *B*” is either a causal effect “*A* → *B*”, or a common cause “*A* ↔ *B*”; “*A* $\circ$ −$\circ$ *B*” (“*A* $\circ$ − $\circ$ *B*”) is either a causal effect “*A* → *B*” or “*A* ← *B*”, a selection bias “*A* − *B*” or a common cause “*A* ↔ *B*”. No edge between variables means that these variables are conditionally independent given the other variables in the network. Reliability estimates for the presence of an edge are depicted as percentages. Direct links between ASD, ADHD, and IQ are marked in *red*. (Color figure online)

Running the BCCD algorithm we inferred reliability estimates of the causal relations between variables (Supplementary Tables S1, S2, S3) and built a graph summarizing these relationships presented in Fig. 1. In this graph an edge between two variables suggests that no other variable in the model can make these variables independent, which we call here a direct relationship. This can be either an effect of one variable on another (“*A* → *B*”), unobserved common cause (“*A* ↔ *B*”) or a selection bias (“*A* − *B*”). If the direction of an edge between two variables is uncertain it has a circle mark “$\circ$”.

The general structure of the network matches other studies in the literature: gender influences symptom counts with males having higher scores than females (Cantwell 1996; Ramtekkar et al. 2010); age influences hyperactivity level with older subjects having lower level of hyperactivity than younger subjects (Biederman et al. 2000); ADHD symptoms are associated with ASD symptoms (Ronald et al. 2008) and both are associated with IQ (with children having ASD, ADHD, or both having lower IQs in general than children without the disorder) (Vaida et al. 2013). Moreover, both ASD and ADHD symptoms are strongly interconnected, resulting in a separate cluster (also called a clique: a complete subgraph, in which all variables are pairwise interconnected) of ADHD and ASD symptoms. The same holds for IQ.

The inferred network suggests that the ASD traits ‘social ineptness’ and ‘stereotyped, repetitive behaviors’ are directly and differentially associated with ADHD symptoms. Social ineptness is associated with inattention and impulsivity, while stereotyped, repetitive behavior is associated with hyperactivity (but not impulsivity). Our network also shows that verbal IQ is a linking factor between ADHD and ASD, since there is a link from verbal IQ to both ADHD and ASD symptom traits. To get a better understanding of these observed direct associations, we zoom in on each link. The direction of these causal links contains circle marks, indicating uncertainty in the causal directions. For example, a link ‘$\circ$−’ between impulsivity and social ineptness and between hyperactivity and repetitive behavior is either a causal link or a selection bias. A link ‘$\circ$→’ between inattention and social ineptness is either a causal link or an unobserved common cause.

**Fig. 2:**
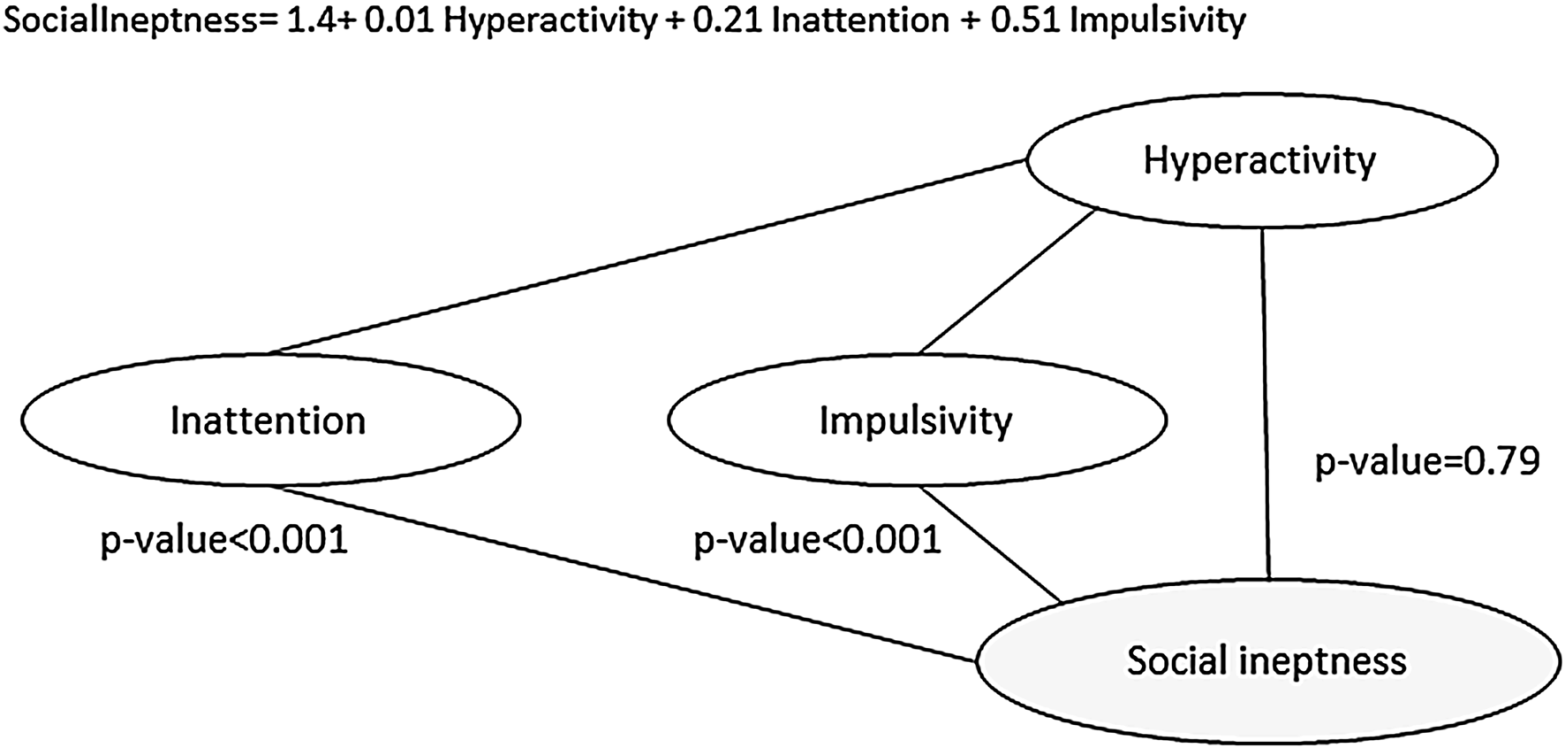
Regression model for mediation analysis that predicts dependent variable (in *grey*) social ineptness using inattention and impulsivity as a mediator and hyperactivity as independent predictor. The regression model is presented at the* top* of the figure, the significance of the regression coefficient is shown next to the edge

Based on the inferred model, there is a direct association of the social ineptness with inattention and impulsivity. Both links have a very strong reliability for a direct link (>99%), providing strong evidence of a direct association. Mediation analysis confirmed that there was no direct link between hyperactivity and social ineptness (β = 0.01, *p* = 0.79) (Fig. 2). We provide the first figure of the regression analysis as an example in the main text, other figures of this type of analysis can be found in the Supplementary material.

Another direct association between ASD and ADHD traits can be seen between hyperactivity and stereotyped, repetitive behaviors (reliability for direct link >99%). No direct causal links are found between repetitive behavior and inattention or impulsivity (Fig. 1). Mediation analysis confirmed that there are no direct paths between inattention and repetitive behavior (β = 0.03, *p* = 0.11), and impulsivity and repetitive behavior (β = 0.01, *p* = 0.86), but an indirect one mediated through social ineptness, which may explain the correlations observed between these variables (Supplementary Fig. S1).

Our analyses also indicated that inattention and social ineptness are associated via verbal IQ due to direct links between inattention and IQ (reliability for direct link >79%), and between social ineptness and IQ (reliability for direct link >99%). Taking into account the link between inattention and social ineptness mentioned before, all three variables are pairwise connected, which can be a sign of an unobserved common cause for these variables. Mediation analysis showed that verbal IQ is only indirectly associated with impulsivity (β = 0.09, *p* = 0.74) and hyperactivity (β =−0.25, *p* = 0.16) and that this is mediated through inattention (Supplementary Fig. S2). Mediation analysis also revealed that there is no direct link between verbal IQ and repetitive behavior (β =−0.27, *p* = 0.12), reduced contact (β = 0.02, *p* = 0.87), and fear of change (β = 0.04, *p* = 0.89), but that these effects are mediated through social ineptness and hyperactivity (Supplementary Fig. S3).

Our model makes preliminary predictions about the directions of the causal links between ASD and ADHD traits. According to the model, hyperactivity may have a causative effect on repetitive behavior, with reliability of the link direction >91%. The direction of this link is inferred from (1) the assumption that hyperactivity does not cause age, and (2) the dependency between age and stereotypic behavior became insignificant when controlling for hyperactivity (R =−0.01, *p* = 0.86). Moreover, our model indicates that impulsivity may have a causative effect on social ineptness (and not visa-versa) with reliability >85%. This direction is inferred from (1) the assumption that impulsivity does not cause age, and (2) the dependency between age and social ineptness became insignificant when controlling for impulsivity (R = 0.01, *p* = 0.79).

## Discussion

In the current study we applied exploratory causal modeling to investigate the co-occurrence of ADHD and ASD by incorporating their core symptom domains into a single integral model. Since ASD and ADHD symptom domains are all significantly pairwise correlated, raw correlation-based methods would not provide any insight into the direct and indirect association between these symptom domains. The causal method applied in this paper builds a more complete model, distinguishes between direct and indirect associations, and allows us to make preliminary predictions about causation. These predictions were corroborated by mediation analysis. The results suggest at least three separate pathways between ADHD and ASD: (a) a pathway from impulsivity to social ineptness, and (b) a pathway from hyperactivity to stereotyped behavior (c) a cluster of inattention, social ineptness and verbal IQ, with a possible common cause.

Our findings suggest that there are multiple distinct pathways and causes for the co-occurrence between ASD and ADHD. The strongest link was found between social communication difficulties, inattention and impulsivity. This corroborates previous reports based on both cross-sectional (Polderman et al. 2014) and longitudinal data (St Pourcain et al. 2014) that part of the association between ASD and ADHD may be due to shared attention-related problems. This is also in accordance with the outcome of reviews by our group (Visser et al. 2016) as well as others (Jones et al. 2014) that attentional problems at a very early age may precede the onset of clinical manifestations of ASD, ADHD, or both disorders. These attentional problems may include, for example, problems in attentional shifting and disengaging impairments (Jones et al. 2014; Visser et al. 2016). As a novel finding, our model putatively suggests that impulsivity has a causative effect on social ineptness. Such a causal link from impulsivity to social ineptness would make intuitive sense. To interact effectively with others, an individual must be able to control impulsive behaviors. Impulsive symptoms may lead a person to miss social cues, for example, because they act prematurely or interrupt the other person (Leitner 2014), which in turn may result in social difficulties. The relevance of impulsivity is reflected in cognitive studies that describe deficits in executive functioning in young children with ADHD and/or ASD, as measured in tests of response inhibition and interference control.

Our model does not make (preliminary) predictions on the causal direction between inattention and social ineptness. It does put inattention and social ineptness in one cluster with verbal IQ, which can be an indication of an unobserved common cause between these variables, for example a shared genetic factor. Verbal IQ refers to the capacity to use language in order to express oneself, comprehend stories, and understand other people, but also to self-directed speech that supports self-control. Previous studies have reported on language problems in both ASD and ADHD (Geurts and Embrechts 2008; Geurts et al. 2004; Leonard et al. 2011). Children with ASD often have a delayed development of spoken language, fail in normal back-and forth conversations, and use language in a stereotypic and repetitive manner. The diagnostic criteria for ADHD also include behaviors suggesting social-communication dysfunction, such as talking excessively, interrupting others, and not listening to what is being said (American Psychiatric Association 2013). These communication deficiencies may contribute to social interaction problems that are typical for individuals with ASD and ADHD. A number of studies have reported on chromosomal regions that may harbor quantitative trait loci (QTLs) for language and communication problems in ASD, including chromosome 7q (Alarcon et al. 2002), which was also identified in a study looking for potential pleiotropic loci for ASD and ADHD (Nijmeijer et al. 2010). Nijmeijer et al. (2010) also found suggestive linkage on chromosome 15q for the SCQ communication subscale in their sample of ADHD families. Furthermore, relatively poor verbal comprehension is more often found in children with ASD (Charman et al. 2011; Rundblad and Annaz 2010). Further study is needed to increase our knowledge on possible pleiotropic (genetic) risk factors that underlie the complex associations between inattention, social ineptness, and verbal competence.

A second pathway identified was between hyperactivity and repetitive behavior. In most studies, impulsivity and hyperactivity are regarded as one combined feature, but our results suggest that these symptoms may be differentially associated with ASD symptoms. Some studies have previously reported on the link between repetitive behaviors and hyperactivity (Polderman et al. 2014, 2013; Gabriels et al. 2005; Ronald et al. 2014). It has been argued that repetitive behavior and ADHD are due to a lack of inhibitory control, but contrasting findings have also been reported (Rommelse et al. 2011). Our model putatively suggests that individuals who are hyperactive and therefore less able to inhibit motor behaviors may, as a result, engage also more often in various motor behaviors that are classified as stereotypic, such as flapping arms/hand when excited or making odd and fast movements with fingers or hands (all items from the CSBQ ‘stereotypic behavior’ subscale). However, Polderman and colleagues (Polderman et al. 2014) proposed that the association may be conversely explained by repetitive behaviors interfering with the ability to switch attention from one task to another. Furthermore, inhibitory control is also associated with impulsivity, which was only indirectly related to repetitive behaviors according to our model. Further research on the direction of the link between hyperactivity and repetitive behavior is therefore needed.

Our putative predictions about the causal directions in the two pathways between ADHD and ASD (from ADHD inattention/impulsivity to ASD social ineptness, and from ADHD hyperactivity to ASD stereotyped, repetitive behavior) suggest that interventions that decrease inattention/impulsivity related difficulties are also likely to have a beneficial effect on social functioning, but not the other way around; interventions that affect social functioning cannot be expected to also have a positive effect on the level of inattention/impulsivity. The same logic is applicable for the effect of hyperactivity on repetitive behavior. These findings are consistent with results from longitudinal study by St. Pourcain et al. (2011). They showed that children with high probability for persistent hyperactive-inattentive symptoms had a high probability for persistent social communication deficits, but not vice versa (St Pourcain et al. 2011). Our results may also fit well with the gradient overarching disorder theory, which proposes that ADHD is a less severe subtype within the ASD spectrum (van der Meer et al. 2012). As a consequence, individuals with (more severe forms of) ADHD are also highly likely to have increased (sub)clinical levels of ASD symptoms. It is important to note however, that our findings are based on just one (albeit rather large) sample, which needs to be replicated in other, independent samples, ideally in a longitudinal design.

Several strengths and limitations should be taken into account when assessing the results of the current study. The main strength of this study is the application of a novel causal discovery method for data analysis. This method considers all variables together, infers both direct and indirect dependencies between variables, provides a reliability measure for each edge in the network, and is able to detect latent common causes. This method does not require longitudinal or interventional data and can infer causal statements based on cross-sectional data (Pearl 2000). Another strength is the use of a large, carefully phenotyped sample of affected and unaffected siblings and control children, allowing us to study the full spectrum of ASD and ADHD symptoms. A limitation of our causal discovery method is that it is an exploratory analysis—it provides new hypotheses that need to be tested using other methods and requires independent replication through experiments or additional data. Another limitation of our study is that the conclusions mainly apply to individuals with average IQ, as we excluded participants with an IQ below 70. This is not representative of the ASD population at large that includes a considerable proportion of individuals with ASD with an intellectual disability. Furthermore, we excluded individuals with known epilepsy, brain disorders, or genetic syndromes, and who were not of European Caucasian descent. Thus, caution is warranted when interpreting our results. In addition, including data of genetically related individuals may cause interpretation problems, due to the possibility of unobserved latent associations between variables that were not taken into account. We tackled this problem by running a sensitivity analysis using a subsample of singletons - including only one subject per family - to evaluate the impact of familiarity. We obtained a highly similar network structure with only a few missing edges due to reduced statistical power as a consequence of the reduction of the sample size by half, indicating the robustness of our approach and our findings.

In conclusion, our results indicate that the often reported co-occurrence of ASD and ADHD might be explained by three distinct pathways: (a) between inattention/impulsivity and social ineptness, and (b) between hyperactivity and stereotypic, repetitive behaviors (c) through verbal IQ. These findings may inform future studies on understanding the (pathophysiological) mechanisms behind the overlap between ASD and ADHD.

## Electronic supplementary material

Below is the link to the electronic supplementary material.

Supplementary material 1 (DOCX 17 KB)

Supplementary material 2 (DOCX 267 KB)
