# Supplementary material for: A Causal and Mediation Analysis of the Comorbidity Between Attention Deficit Hyperactivity Disorder (ADHD) and Autism Spectrum Disorder (ASD)
Source: J Autism Dev Disord. 2017 Mar 2;47(6):1595–604. doi: 10.1007/s10803-017-3083-7 (PMC5432632; doi:10.1007/s10803-017-3083-7)
Supplement: Supplementary file 1 — Supplementary material 1 (DOCX 17 KB) [file 10803_2017_3083_MOESM1_ESM.docx]

| ‘**Reduced contact and social interest’ (*Social*, 12 items, α = .82):** |
| --- |
| 20 Has little or no need for contact with others |
| 16 Makes little eye contact |
| 18 Does not seek comfort when he/she is hurt or upset |
| 17 Dislikes physical contact e.g., does not want to be touched or hugged |
| 21 Does not respond to initiatives by others e.g., does not play along when asked |
| 19 Does not initiate play with other children |
| 14 Acts as if others are not there |
| 15 Lives in a world of his/her own |
| 34 Does not show his/her feelings in facial expressions and/or bodily posture |
| 13 Does not look up when spoken to |
| 33 Cannot be made enthusiastic about anything; does not particularly like anything |
| 41 Does not appreciate it when someone else is hurt or sad |
| **‘Difficulties in understanding social information’ (*Understanding*, 7 items, α = .82):** |
| 6 Takes things literally e.g., does not understand certain expressions |
| 5 Does not understand jokes |
| 3 Does not fully understand what is being said to him/her i.e., tends to miss the point |
| 7 Is exceptionally naive; believes anything you say |
| 4 Frequently says things that are not relevant to the conversation |
| 1 Talks confusedly; jumps from one subject to another in speaking |
| 2 Only talks about things that are of concern for himself/herself |
| **‘Fear of and resistance to changes’ (*Change*, 3 items, α = .83):** |
| 46 Remains clammed up in new situations or if change occurs |
| 45 Panics in new situations or if change occurs |
| 47 Opposes change |
| **‘Stereotyped behavior’ (*Stereotypies*, 8 items, α = .78):** |
| 25 Constantly feels objects |
| 24 Smells objects |
| 11 Makes odd, fast movements with fingers or hands |
| 23 Is extremely pleased by certain movements and keeps doing them e.g., turning around and around |
| 10 Flaps arms/hands when excited |
| 26 Is fascinated by certain colors, forms, or moving objects |
| 12 Sways to and fro |
| 22 Is unusually sensitive to certain sounds e.g., always hears certain sounds earlier than other people |
| ‘Not optimally tuned to the social situation’ (*Tuned*, 11 items, α = .87): |
| 31 Quickly gets angry |
| 43 Does not know when to stop, e.g., goes on and on about things |
| 44 Is extremely stubborn |
| 32 Stays angry for a long time e.g., when he/she does not get his/her way |
| 37 Is disobedient |
| 9 Draws excessive attention to him/herself |
| 30 Shows sudden changes of mood |
| 42 Makes a fuss over little things; ‘‘makes a mountain of a mole-hill’’ |
| 8 Over-reacts to everything and everyone |
| 38 Cannot be corrected in situations in which he/she has done something wrong |
| 40 Makes inconsiderate remarks e.g., remarks that are painful to others |
| ‘Orientation problems in time, place, or activity’ (*Orientation*, 8 items, α = .80): |
| 29 Does things without realizing the aim e.g., constantly has to be reminded to finish things |
| 28 Does things without realizing what stage of the activity he/she has reached (beginning, middle, ending) |
| 49 Has no sense of time |
| 39 Takes in information with difficulty |
| 27 Has difficulties doing two things simultaneously e.g., he/she cannot dress and listen to parent at the same time |
| 35 Does not appreciate danger |
| 48 Gets lost easily e.g., when out with someone |
| 36 Barely distinguishes between strangers and familiar people e.g., readily goes with strangers |

From Greaves-lord et al. (2012).

This publication also explains why it is most insightful to focus on the first four subscales of the CSBQ –like we did- when looking specifically for ASD symptoms (p. 1788, first paragraph)
