# Supplementary material for: A Causal and Mediation Analysis of the Comorbidity Between Attention Deficit Hyperactivity Disorder (ADHD) and Autism Spectrum Disorder (ASD)
Source: J Autism Dev Disord. 2017 Mar 2;47(6):1595–604. doi: 10.1007/s10803-017-3083-7 (PMC5432632; doi:10.1007/s10803-017-3083-7)
Supplement: Supplementary file 2 — Supplementary material 2 (DOCX 267 KB) [file 10803_2017_3083_MOESM2_ESM.docx]

## Supplementary material

**Table S1** Demographics of the study sample. The standard deviation of the average value is indicated in brackets.

|  | Controls | | Siblings | | | | Patients | | |
| --- | --- | --- | --- | --- | --- | --- | --- | --- | --- |
| Row Labels | unaffected | ADHD only | | ASS + ADHD | ASS only | unaffected | ADHD only | ASS + ADHD | ASS only |
| Total N | 414 | 116 | | 24 | 29 | 393 | 201 | 115 | 101 |
| (%) Male | 42% | 59% | | 75% | 62% | 42% | 86% | 83% | 84% |
| Age in years | 11.20 (min= 4.33, max= 20.08) | 10.84 (min=5.47, max=20.2) | | 11.49 (min=7.34, max=17.75) | 11.85 (min=4.08, max=20.08) | 11.57 (min=4.08, max=21.42) | 11.67 (min=5.83, max=17.77) | 11.43 (min=4.17, max=18.83) | 12.20 (min=4.08, max=19.83) |
| Inattentiveness raw score  (minimum level=0, maximum level=27) | 2.54 (2.67) | 13.83 (4.72) | | 14.90 (4.91) | 9.00 (4.02) | 3.91 (3.42) | 17.36 (3.81) | 16.18 (3.99) | 9.54 (4.19) |
| Hyperactivity raw score  (minimum level=0, maximum level=18) | 1.13 (1.32) | 6.71 (3.38) | | 7.27 (2.51) | 4.66 (2.82) | 1.74 (1.87) | 10.07 (2.91) | 8.60 (3.12) | 4.25 (2.47) |
| Impulsivity raw score (minimum level=0, maximum level=9) | 0.63 (0.88) | 3.46 (1.92) | | 4.42 (2.02) | 3.00 (1.73) | 1.05 (1.28) | 5.59 (1.70) | 5.14 (1.94) | 2.67 (1.66) |
| Reduced contact and social interests  (minimum level=0, maximum level=22) | 0.94 (1.72) | 3.52 (3.77) | | 10.62 (4.81) | 8.41 (4) | 2.13 (3.08) | 5.16 (4.02) | 10.24 (5.2) | 11.49 (5.23) |
| Social ineptness  (minimum level=0, maximum level=14) | 1.41 (1.74) | 5.63 (3.54) | | 8.95 (3.57) | 7.35 (3.1) | 2.82 (2.81) | 7.01 (3.61) | 9.36 (3.05) | 8.66 (2.93) |
| Fear of and resistance to changes  (minimum level=0, maximum level=6) | 0.33 (1.04) | 1.49 (3.36) | | 3.24 (3.07) | 3.24 (2.21) | 0.66 (1.63) | 2.2 (3.32) | 3.01 (4.05) | 2.87 (3.26) |
| Repetitive behavior  (minimum level=0, maximum level=15) | 0.45 (0.84) | 2.72 (1.68) | | 5.05 (1.89) | 2.59 (1.35) | 0.84 (1.18) | 4.4 (2) | 6.03 (1.92) | 4.17 (1.92) |
| Verbal IQ | 107.64 (13.16) | 99.39 (13.82) | | 94.43 (15.31) | 101.11 (15.23) | 103.65 (13.42) | 97.40 (12.76) | 93.71 (16.89) | 96.60 (18.46) |
| Performance IQ | 105.82 (14.25) | 102.59 (13.91) | | 101.98 (18.53) | 104.72 (20.11) | 105.45 (14.84) | 101.73 (15.08) | 101.32 (17.89) | 101.74 (18.98) |

**Causal modeling**

One of the most popular and intuitive ways to represent causal models in the social sciences is through structural equation modeling (SEM) (1). One way of working with SEMs is to provide several hypothetical networks based on some prior knowledge and then compare these networks based on a particular metric (for example the AIC or BIC score). This is a confirmatory analysis that is used to test a particular hypothesis, which is often applied in, for example, twin studies. Another approach is to try and learn the structure of SEM from the observed data. The basic idea of structure learning algorithms is described in Turing award winner Judea Pearl’s work (2) that shows a connection between conditional independencies and causal relationships. Thus, by learning conditional independencies in cross-section data, it is possible in particular cases to learn the structure of a SEM and to make predictions about causation. Causal modeling is primarily an exploratory approach aiming to find novel causal paths that were not known in advance. In this paper we consider the second approach, since our goal is to explore links between ADHD and ASD traits rather than to confirm known links.

The two main approaches to learn the structure of a SEM from data are the so-called score-based and constraint-based approaches (2, 3). The score-based approach provides a measure of reliability of the inferred causal network which makes the interpretation of the results easier and prevents incorrect categorical decisions (4). However, this approach often relies on the assumption that there are no common confounders of the observed variables. The constraint-based approach does not have to rely on the assumption that there are no common confounders, and, as a result, can sometimes detect the presence of confounders between observed variables from the data (5). A drawback of this approach is lack of robustness in some cases. Typical implementations makes use of independence tests, making the results for borderline independencies/dependencies incorrect sometimes (6). As a result, the outcome of learning a network can be sensitive to such errors. In this study we applied a state-of-the-art algorithm for structure learning called Bayesian Constraint-based Causal Discovery (BCCD) (7) to infer the causal structure from the data. BCCD combines the strength of constraint-based and score-based approaches, which allows it to outperform the best algorithms in the field (7). This algorithm is able to detect common causes of the observed variables similar to constraint-based approaches and provides a reliability measure of the inferred relationship like the score-based approach. This reliability measure gives a conservative estimate of the probability of a causal relation. A recently extended version of BCCD can handle data that contains a mixture of discrete and continuous variables and missing values and does not require discretization or imputation that can lead to loss of information or biased results (8).

BCCD can handle directed acyclic graphs that contain latent variables. These graphs are called maximal ancestral graphs (MAG). All MAGs that represent the same set of conditional independencies form an equivalence class. The equivalence class for MAGs is called a partial ancestral graph (PAG). The BCCD algorithm produces PAGs as an output. An edge between two variables in PAG suggests that there is a direct causal relationship between them. This can be either an effect of one variable on another (“$A \to B$”), unobserved common cause “$A \leftrightarrow B$” or a selection bias “$A-B$”. If a direction of an edge between two variables is non-identifiable it is marked with a circle mark “○”. No edge between variables means that these variables are conditionally independent given other variables in the network. For example, if $A$ and $B$ are correlated but there is no edge between them in the PAG, that implies that there is either an indirect causal path from one variable to another through some other variables in this PAG, a common cause between these variables, or a selection bias.

**Results BCCD**

Running the BCCD algorithm provided three tables (Supplementary material Tables S2, S3, and S4). Table S2 provides the reliability of the statement that a direct link exists between two variables. Table S3 presents the reliability of the causal statement: “$A$ causes $B$”, both for direct and indirect causal effects. If Table S3 says that “$A$ causes $B$” and there is an edge between $A$ and $B$, this edge has a tail from $A$ to $B$ and $A$ causes $B$ in the PAG. For example, the variable “Gender” caused variable “Inattention” with reliability 51%. Table S3 represents the reliability of the causal statement: “$A$ does not cause $B$”, both for direct and indirect causal effects. If Table S4 says that “$A$ does not cause $B$” and there is an edge between $A$ and $B$, this edge has an arrow head from $B$ to $A$. For example, the variable “PIQ” does not cause variable “VIQ” with reliability 53%. The prior knowledge used in the model, e.g. that gender is not caused by other variables and, is represented in Table S3 in cells with a reliability of 100%.

The difference between Tables S2 and S3 compared to Table S4 is that Tables S3 and S4 give an estimate for the direction of the causal effect, whereas Table S2 provides estimates for the presence of the edge between two variables. Moreover, Tables S3 and S4 show the reliability of the direction of both direct and indirect causal paths, whereas Table S2 gives a reliability estimate for a direct causal path between two variables only.

**Table S2.** Reliability of direct links between two variables.

|  | Age | Gender | Reduced contact | Social ineptness | Repetitive behavior | Fear of changes | Verbal IQ | Performance IQ | Inattention | Hyperactivity | Impulsivity |
| --- | --- | --- | --- | --- | --- | --- | --- | --- | --- | --- | --- |
| Age | - | 0.00 | 0.90 | 0.08 | 0.04 | 0.03 | 0.54 | 0.04 | 0.03 | 0.71 | 0.14 |
| Gender | - | - | 0.90 | 0.06 | 0.28 | 0.03 | 0.08 | 0.11 | 1.00 | 0.25 | 0.43 |
| Reduced contact | - | - | - | 1.00 | 1.00 | 1.00 | 0.04 | 0.04 | 0.31 | 0.04 | 0.04 |
| Social ineptness | - | - | - | - | 1.00 | 1.00 | 1.00 | 0.05 | 1.00 | 0.25 | 1.00 |
| Repetitive behavior | - | - | - | - | - | 1.00 | 0.08 | 0.05 | 0.05 | 1.00 | 0.44 |
| Fear of changes | - | - | - | - | - | - | 0.04 | 0.18 | 0.07 | 0.06 | 0.10 |
| Verbal IQ | - | - | - | - | - | - | - | 1.00 | 0.79 | 0.07 | 0.04 |
| Performance IQ | - | - | - | - | - | - | - | - | 0.05 | 0.04 | 0.04 |
| Inattention | - | - | - | - | - | - | - | - | - | 1.00 | 1.00 |
| Hyperactivity | - | - | - | - | - | - | - | - | - | - | 1.00 |
| Impulsivity | - | - | - | - | - | - | - | - | - | - | - |

**Table S3** The reliability estimate of the logical statement “$A$ causes $B$*”*, where $A$ is represented in rows and $B$ in columns. The estimate is provided for logical statements with reliability of 50% or higher.

|  | Age | Gender | Reduced contact | Social ineptness | Repetitive behavior | Fear of changes | Verbal IQ | Performance IQ | Inattention | Hyperactivity | Impulsivity |
| --- | --- | --- | --- | --- | --- | --- | --- | --- | --- | --- | --- |
| Age | - | - | 0.59 | 0.63 | - | - | 0.83 | 0.68 | - | 0.63 | - |
| Gender | - | - | 0.69 | 0.69 | - | 0.77 | - | - | 0.77 | 0.66 | - |
| Reduced contact | - | - | - | - | - | 0.99 | - | - | - | - | - |
| Social ineptness | - | - | - | - | - | - | - | - | - | - | - |
| Repetitive behavior | - | - | - | - | 0.82 | - | - | - | - | - | 0.80 |
| Fear of changes | - | - | - | - | - | - | - | - | - | - | - |
| Verbal IQ | - | - | - | - | - | - | - | - | - | - | - |
| Performance IQ | - | - | - | - | - | - | - | - | - | - | - |
| Inattention | - | - | - | - | - | - | - | - | - | - | - |
| Hyperactivity | - | - | - | - | 0.91 | - | 0.78 | - | - | - | 0.81 |
| Impulsivity | - | - | - | 0.85 | - | - | - | - | - | - | - |

**Table S4**. The reliability of the logical statement “$A$ does not cause $B$*”,* where $A$ is represented in rows and $B$ in columns. The estimate is provided for logical statements with reliability of 50% or higher. Cells with a reliability of 100% represent the prior knowledge used in the model, e.g. that gender is not caused by other variables.

|  | Age | Gender | Reduced contact | Social ineptness | Repetitive behavior | Fear of changes | Verbal IQ | Performance IQ | Inattention | Hyperactivity | Impulsivity |
| --- | --- | --- | --- | --- | --- | --- | --- | --- | --- | --- | --- |
| Age | - | 1.00 | - | - | 0.77 | 0.77 | - | - | 0.76 | - | - |
| Gender | 1.00 | - | - | - | - | - | - | 0.87 | - | - | - |
| Reduced contact | 1.00 | 1.00 | 0.98 | 0.98 | 0.98 | - | - | 0.97 | - | 0.97 | 0.95 |
| Social ineptness | 1.00 | 1.00 | - | 0.96 | - | - | - | - | 0.96 | - | - |
| Repetitive behavior | 1.00 | 1.00 | - | - | - | - | - | - | - | - | - |
| Fear of changes | 1.00 | 1.00 | 0.99 | 0.84 | 0.98 | 0.99 | - | - | - | - | - |
| Verbal IQ | 1.00 | 1.00 | 0.89 | 0.88 | 0.83 | 0.88 | 0.89 | - | 0.82 | 0.86 | 0.80 |
| Performance IQ | 1.00 | 1.00 | 0.74 | 0.73 | 0.59 | 0.73 | 0.74 | 0.74 | - | 0.71 | 0.66 |
| Inattention | 1.00 | 1.00 | 0.93 | - | - | - | - | 0.88 | 0.93 | - | - |
| Hyperactivity | 1.00 | 1.00 | 0.88 | - | - | - | - | - | 0.96 | 0.96 | - |
| Impulsivity | 1.00 | 1.00 | 0.86 | - | 0.80 | - | - | - | - | - | 0.86 |

**Mediation analysis**

We applied mediation analysis to investigate whether there is a direct link between hyperactivity trait of ADHD and social ineptness trait of ASD. We built a regression model where social ineptness trait is a dependent variable, inattention and impulsivity are possible mediators and hyperactivity is an independent variables. Regression analysis showed that hyperactivity is not a significant predictor of social ineptness ($\beta$=0.01, p=0.79). Thus, we can conclude that there is no direct link between hyperactivity and social ineptness.

Using mediation analysis we tested whether there is an evidence of no direct link between inattention and repetitive behavior as well as between impulsivity and repetitive behavior. We built a regression model where repetitive behavior is a dependent variable, social ineptness, and hyperactivity are possible mediators, and inattention (Figure S1a) or impulsivity (Figure S1b) are independent variables. Analysis showed that the regression coefficients are not significant between inattention and repetitive behavior ($\beta$=0.03, p=0.11), and between impulsivity and repetitive behavior ($\beta$=0.01, p=0.86).


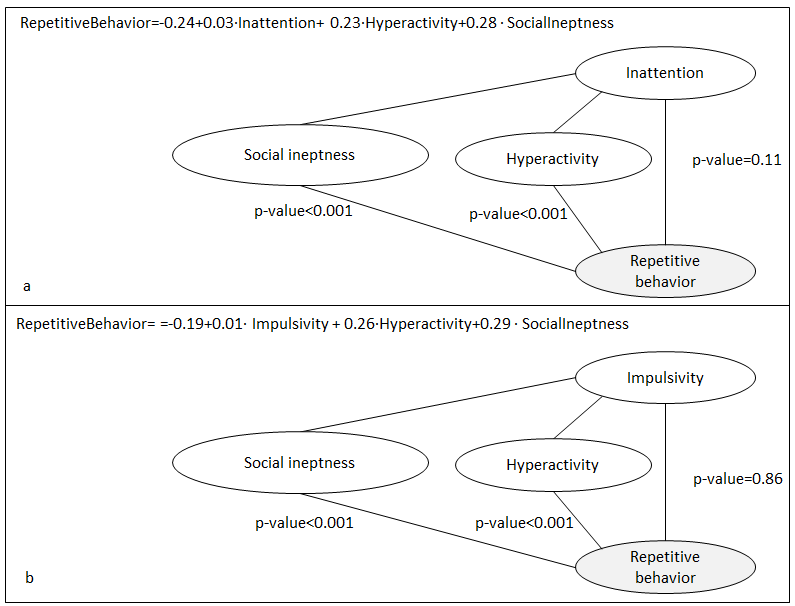


Figure S1. Regression model for mediation analysis that predicts dependent variable (in grey) repetitive behavior using, social ineptness, and hyperactivity as a mediator and inattention (a) or impulsivity (b) as independent predictor. The regression model is presented at the top of the figure, the significance of the regression coefficient is shown next to the edge.

We investigated whether the direct and indirect associations between verbal IQ with ADHD and ASD symptoms described above can be explained with mediation analysis. First we explored whether verbal IQ is associated directly only with one ADHD trait (inattention). To check this hypothesis, we built a regression model, where verbal IQ is a dependent variable, inattention is a possible mediator, and hyperactivity (Figure S2a), and impulsivity (Figure S2b) are independent variables. Our analysis showed that neither hyperactivity ($\beta$=-0.25, p=0.16) nor for impulsivity ($\beta$=0.09, p=0.74) are significant predictor for verbal IQ when inattention trait is present in the regression model. This confirms that the association between verbal IQ and impulsivity as well as hyperactivity is not direct and mediated through inattention.


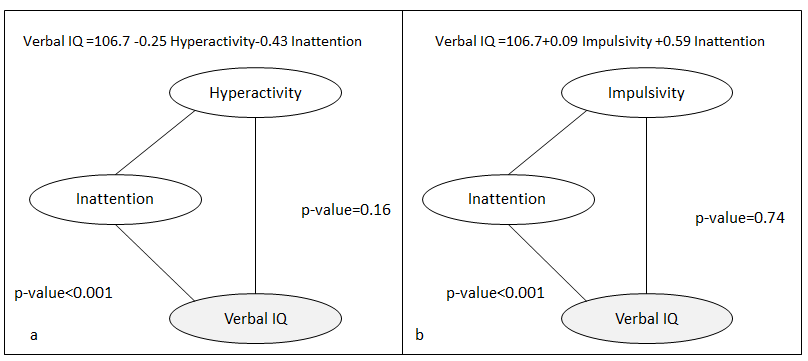


Figure S2. Regression model for mediation analysis that predicts dependent variable (in grey) verbal IQ using inattention as a mediator and hyperactivity (a) or impulsivity (b) as independent predictor. The regression model is presented at the top of the figure, the significance of the regression coefficient is shown next to the edge.

Then we investigated whether verbal IQ is not directly associated with repetitive behavior, reduced contact and fear of changes trait of ASD as was shown by BCCD. We built a regression model where verbal IQ is a dependent variable, social ineptness and hyperactivity(only for model with repetitive behavior) are possible mediators, and repetitive behavior (Figure 3a), reduced contact (Figure 3b), and fear of changes (Figure 3c) are an independent variable. Mediation analysis showed that the regression coefficients of repetitive behavior ($\beta$=-0.27, p=0.12), reduced contact ($\beta$=0.02, p=0.87) and fear of changes ($\beta$=0.04, p=0.89) are not significant in such a model, suggesting that there is no direct effect between these variables and verbal IQ and, but this effect is mediated via the other traits. This confirms our previous findings with the BCCD algorithm.


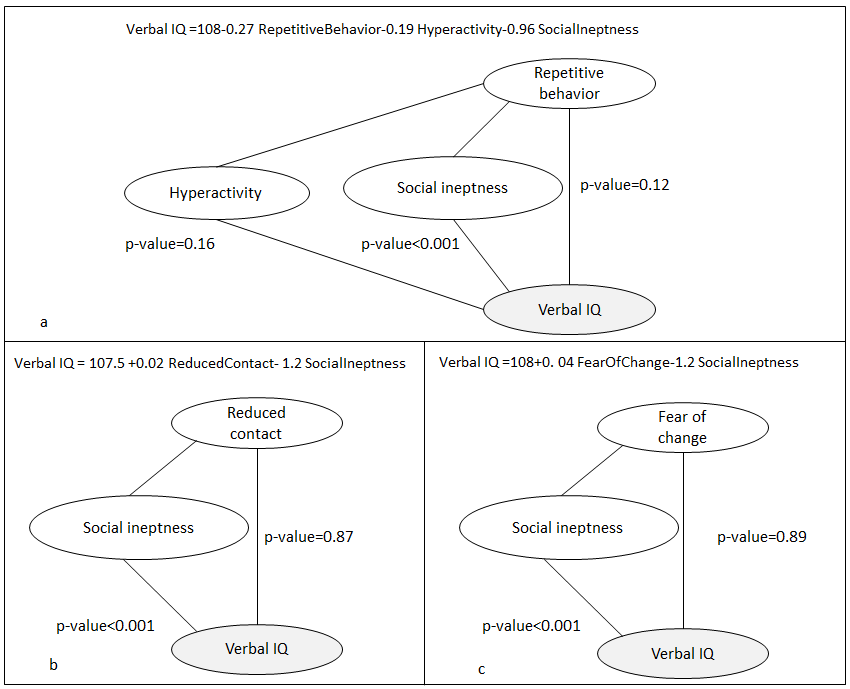


Figure S3. Regression model for mediation analysis that predicts dependent variable (in grey) verbal IQ using social ineptness (and hyperactivity in (a)) as a mediator and repetitive behavior (a), reduced contact (b), or fear of changes (c) as independent predictor. The regression model is presented at the top of the figure, the significance of the regression coefficient is shown next to the edge.


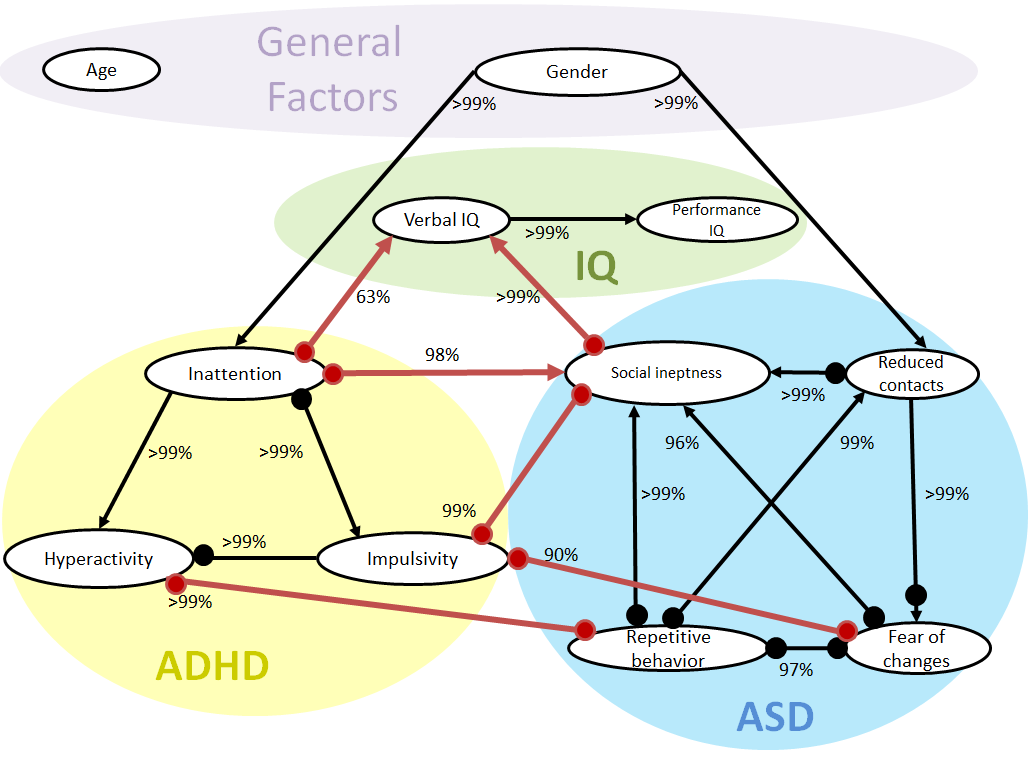


Figure S4. Output causal model representing causal relationships between variables in the ADHD ASD data set when reduced to one subject per family. Edge directions are marked with ‘-’ and ‘ >’ for identifiable edge directions and with ‘○’ for non- identifiable edge directions. Reliability estimates for the presence of an edge are depicted as percentage. Direct links between ASD, ADHD and IQ are marked in red.

## References

1. Beran TN, Violato C. Structural equation modeling in medical research: a primer. BMC Res Notes. 2010;3:267.

2. Pearl J. Causality: models, reasoning and inference: Cambridge University Press; 2000.

3. Daly R, Shen Q, Aitken S. Learning Bayesian networks: approaches and issues. Knowl Eng Rev. 2011;26(2):99-157.

4. Heckerman D, Meek C, Cooper G. A Bayesian approach to causal discovery. Computation, causation, and discovery. 1999;19:141-66.

5. Spirtes P, Glymour CN, Scheines R. Causation, prediction, and search: MIT press; 2000.

6. Spirtes P. Introduction to causal inference. J Mach Learn Res. 2010;11:1643-62.

7. Claassen T, Heskes T, editors. A Bayesian approach to constraint based causal inference. Proceedings of the 28th Conference on Uncertainty in Artificial Intelligence 2012.

8. Sokolova E, Groot P, Claassen T, von Rhein D, Buitelaar J, Heskes T. Causal discovery from medical data: dealing with missing values and a mixture of discrete and continuous data. Proceedings of the 15th Conference on Artificial Intelligence in Medicine: Springer; 2015. p. 177-81.
